# *Weissella viridescens* Attenuates Hepatic Injury, Oxidative Stress, and Inflammation in a Rat Model of High-Fat Diet-Induced MASLD

**DOI:** 10.3390/nu17091585

**Published:** 2025-05-05

**Authors:** Shuwei Zhang, Ruiqing Zhao, Ruoshi Wang, Yao Lu, Mingchao Xu, Xiaoying Lin, Ruiting Lan, Suping Zhang, Huijing Tang, Qianhua Fan, Jing Yang, Liyun Liu, Jianguo Xu

**Affiliations:** 1 School of Public Health, Nanjing Medical University, Nanjing 211166, China; zhangshuweisc@163.com (S.Z.);; 2 National Key Laboratory of Intelligent Tracking and Forecasting for Infectious Diseases, National Institute for Communicable Disease Control and Prevention, Chinese Center for Disease Control and Prevention, Beijing 102206, China; 3 Department of Epidemiology and Statistics, School of Public Health, Hebei Medical University, Shijiazhuang 050010, China; 4 School of Biotechnology and Biomolecular Sciences, University of New South Wales, Sydney, NSW 2052, Australia; 5 Research Units of Discovery of Unknown Bacteria and Function, Chinese Academy of Medical Sciences, Beijing 102206, China; 6 Hebei Key Laboratory of Intractable Pathogens, Shijiazhuang Center for Disease Control and Prevention, Shijiazhuang 050011, China

**Keywords:** *Weissella viridescens*, metabolic-dysfunction-associated steatotic liver disease, inflammation, oxidative stress, gut microbiota

## Abstract

**Background:** Metabolic-dysfunction-associated steatotic liver disease (MASLD) is the most prevalent chronic liver disorder globally. Probiotic supplementation has shown promise in its prevention and treatment. Although *Weissella viridescens*, a lactic acid bacterium with immunomodulatory effects, has antibacterial and anti-inflammatory activities, there is a lack of direct evidence for its role in alleviating MASLD. This study aimed to investigate the protective effects of *W. viridescens* strain Wv2365, isolated from healthy human feces, in a high-fat diet (HFD)-induced rat model of MASLD. **Methods:** Rats were randomly assigned to a normal chow diet (NC), high-fat diet (HFD), and HFD supplemented with *W. viridescens* Wv2365 (Wv2365) groups. All groups were fed their respective diets for 8 weeks. During this period, the NC and HFD groups received a daily oral gavage of PBS, while the Wv2365 group received a daily oral gavage of Wv2365. **Results:** Wv2365 supplementation significantly reduced HFD-induced body weight gain, improved NAFLD activity scores, alleviated hepatic injury, and restored lipid metabolism. A liver transcriptomic analysis revealed the downregulation of inflammation-related pathways, along with decreased serum levels of TNF-α, IL-1β, IL-6, MCP-1, and LPS. Wv2365 also activated the Nrf2/HO-1 antioxidant pathway, enhanced hepatic antioxidant enzyme activities and reduced malondialdehyde levels. A gut microbiota analysis showed the enrichment of beneficial genera, including *Butyricicoccus*, *Akkermansia*, and *Blautia*. Serum metabolomic profiling revealed increased levels of metabolites including indole-3-propionic acid, indoleacrylic acid, and glycolithocholic acid. **Conclusions:** Wv2365 attenuates hepatic injury, oxidative stress, and inflammation in a rat model of high-fat-diet-induced MASLD, supporting its potential as a probiotic candidate for the modulation of MASLD.

## 1. Introduction

Non-alcoholic fatty liver disease (NAFLD), recently renamed metabolic-dysfunction-associated steatotic liver disease (MASLD) [1], is the most prevalent chronic liver disease worldwide, affecting approximately 30% of the global population [2,3,4]. It is characterized by excessive hepatic lipid accumulation, oxidative stress, and inflammation [5,6]. In recent years, advancements in the understanding of MASLD pathogenesis have led to a shift from the traditional “two-hit” hypothesis to the more comprehensive “multiple-hit” hypothesis [7,8], which emphasizes the critical role of the gut–liver axis in the onset and progression of the disease [9,10]. Increasing evidence indicates that the gut microbiota and its metabolites are critically involved in modulating hepatic inflammation and oxidative damage [11,12,13,14]. In addition, dietary interventions have been recognized as an essential component in the prevention and management of MASLD by modulating metabolic risk factors and liver inflammation [15]. As a dietary approach to modulating the gut microbiota, probiotic supplementation has emerged as a promising strategy for the prevention and treatment of MASLD [8,12,16], among which lactic acid bacteria (LAB) have attracted considerable attention due to their probiotic potential [17,18,19,20].

The genus *Weissella*, belonging to the phylum Firmicutes, class Bacilli, order Lactobacillales, and family Leuconostocaceae, is widely distributed in fermented foods and the gastrointestinal tracts of humans and animals [21]. Although *Weissella* is relatively new compared with other LAB genera, recent studies have demonstrated its probiotic potential, including multiple beneficial effects and the ability to produce exopolysaccharides [20], suggesting its promising applications in both pharmaceutical and food production sectors [22]. Among the species of *Weissella*, *W. viridescens* has attracted particular attention because of its significant antimicrobial and anti-inflammatory properties [23,24]. For instance, *W. viridescens* has been reported to exert significant inhibitory effects against Listeria monocytogenes [25]. Furthermore, *W. viridescens* UCO-SMC3 has been demonstrated to possess immunomodulatory effects via the upregulation of the interleukin-10 (IL-10) pathway, thereby alleviating inflammation associated with acne [26]. These findings collectively suggest that *W. viridescens* may confer probiotic benefits through its antimicrobial and anti-inflammatory activities. However, despite these promising attributes, direct evidence for the beneficial effects of *W. viridescens* on MASLD remains limited.

This study was designed to evaluate the protective effects of *W. viridescens* Wv2365, a strain isolated from the feces of a healthy individual, using an HFD-induced MASLD rat model. Specifically, we investigated the impact of Wv2365 on hepatic lipid accumulation, oxidative stress, inflammation, gut microbiota composition, and serum metabolism. The findings of this study provide experimental evidence supporting Wv2365 as a potential probiotic for improving MASLD symptoms and offer new insights into microbiota-based therapeutic strategies for MASLD.

## 2. Materials and Methods

### 2.1. Isolation of Strains and Growth Conditions

*Weissella viridescens* Wv2365 was isolated from the feces of a healthy adult and stored in our laboratory [27]. The strain was submitted to the China General Microbiological Culture Collection Center (CGMCC) and assigned the accession number CGMCC 27140. It was anaerobically cultured at 37 °C for 18–24 h on De Man, Rogosa, and Sharpe (MRS) medium (OXOID, Lenexa, KS, USA) supplemented with 5% (*v*/*v*) defibrinated sheep blood.

### 2.2. Animals Model

Normal diet (NC, 10% calories from fat, D12450J) and high-fat diet (HFD, 60% calories from fat, D12492) were purchased from Research Diets Inc. (New Brunswick, NJ, USA). All animal experiments were approved and conducted in accordance with the guidelines of the Ethics Review Committee of the National Institute for Communicable Disease Control and Prevention, Chinese Center for Disease Control and Prevention (Approval No. 2022-036). Specific-pathogen-free (SPF) Wistar rats (Male; 180 ± 20 g) were obtained from Vital River Lab Animal Technology Co., Ltd. (Beijing, China). All rats were housed under controlled conditions (12 h light/dark cycle, 23 ± 2 °C, and 55 ± 5% humidity) with ad libitum access to food and water. After one week acclimation period, the rats were randomly assigned into three groups (NC, HFD, and HFD + Wv2365). Rats in the HFD + Wv2365 group were fed HFD and intragastrically administered *W. viridescens* Wv2365 (1.0 × 10^9^ CFU/mL) for 8 weeks. The NC and HFD groups were administered the corresponding diets and received an equal volume of PBS solution via oral gavage for 8 weeks. Food consumption was recorded weekly to determine individual energy intake, and body weight was measured at the same intervals. Upon completion of the experiment, the rats were euthanized, and blood samples were collected from the abdominal aorta. Serum was obtained by centrifuging the blood at 1500× *g* for 15 min at 4 °C and stored at −80 °C for subsequent analyses. Liver and epididymal fat tissues were excised, weighed, and divided into three parts: one was fixed in 4% paraformaldehyde at room temperature, one was preserved in RNAlater and stored at −80 °C, and one was snap-frozen in liquid nitrogen and then stored at −80 °C.

### 2.3. Biochemical Parameters of Serum and Liver

Serum levels of triglyceride (TG), total cholesterol (TC), high-density lipoprotein cholesterol (HDL-C), low-density lipoprotein cholesterol (LDL-C), alanine aminotransferase (ALT), aspartate aminotransferase (AST), and alkaline phosphatase (ALP) were measured using an automatic biochemical analyzer with commercial kits (R&D Systems, Minneapolis, MN, USA). Protein levels of tumor necrosis factor-alpha (TNF-α), interleukin-1 beta (IL-1β), interleukin-6 (IL-6), monocyte chemoattractant protein-1(MCP-1), leptin, lipopolysaccharide (LPS), free fatty acid (FFA), and hepatic TG were quantified using enzyme-linked immunosorbent assay (ELISA) kits from the same manufacturer. Additionally, the activities of oxidative stress-related enzymes, including superoxide dismutase (SOD), catalase (CAT), glutathione peroxidase (GSH-Px), and heme oxygenase-1 (HO-1), as well as the oxidative damage marker malondialdehyde (MDA), were assessed using ELISA kits.

### 2.4. Histological Analyses

Liver and epididymal fat were fixed in 4% paraformaldehyde, embedded in paraffin, sectioned at 4 µm, and stained with hematoxylin and eosin (H&E). Stained sections were imaged using a bright-field microscope (Nikon Eclipse E100, Nikon Corporation, Tokyo, Japan). These procedures were conducted at Wuhan Servicebio Technology Co., Ltd. (Wuhan, Hubei, China). The severity of hepatic lesions was evaluated using the NAFLD Activity Score (NAS) [28], which comprises three components: steatosis (scored 0–3), lobular inflammation (0–3), and hepatocellular ballooning (0–2).

### 2.5. Transcriptome Analysis

Total hepatic RNA was extracted from 18 rats (6 samples per group) using TRIzol reagent (Life Technologies, Carlsbad, CA, USA). The concentration and purity of extracted RNA were measured with a NanoDrop™ 2000 spectrophotometer (Thermo Fisher Scientific, Wilmington, DE, USA), while RNA integrity was determined using the RNA Nano 6000 Assay Kit on the Agilent 2100 Bioanalyzer system (Agilent Technologies, Santa Clara, CA, USA). RNA libraries were constructed using the NEBNext^®^ Ultra™ RNA Library Preparation Kit (New England Biolabs, Ipswich, MA, USA), and sequencing was performed on the Illumina NovaSeq system, generating 150 bp paired-end reads. Differential expression analysis was performed with DESeq2, and differentially expressed genes (DEGs) were defined as those meeting the criteria: |log2 fold change| > 1 and adjusted *p*-value < 0.05. Data analysis was conducted on the BMK Cloud platform (https://www.biocloud.net, accessed on 9 February 2025).

### 2.6. Real-Time Quantitative PCR (RT-qPCR)

The expression levels of selected genes were validated through real-time quantitative PCR (RT-qPCR). Total RNA was transcribed into complementary DNA (cDNA) using the PrimeScript™ RT reagent kit (Takara, Dalian, China) in accordance with the supplier’s protocol. Amplification was performed using TB Green^®^ Premix Ex Taq™ II (Takara) and specific primers obtained from Sangon Biotech (Shanghai, China). The primer sequences were as follows: *Nrf2* forward 5′-TGGATCTGTCAGCTACTCCCA-3′ and reverse 5′-ATCCAGGGCAAGCGACTCAT-3′; and *Gapdh* (housekeeping gene) forward 5′-GCAAGTTCAACGGCACAG-3′ and reverse 5′-CGCCAGTAGACTCCACGAC-3′. PCR reactions were prepared in a 10 μL system and run according to the kit protocol. The 2^−ΔΔCt method was used to determine the relative mRNA expression levels, normalized to *Gapdh*.

### 2.7. 16S rRNA Sequencing and Gut Microbiota Analysis

Total genomic DNA was extracted from rat fecal samples using the TGuide S96 Magnetic Stool DNA Kit (Tiangen Biotech (Beijing) Co., Ltd, Beijing, China) following the manufacturer’s instructions. The V3–V4 regions of the 16S rRNA gene were amplified and sequenced on the Illumina Novaseq 6000 platform (paired ends, 2 × 250 bp). Qualified sequences were clustered into Amplicon Sequence Variants (ASVs) at a 97% similarity threshold using USEARCH (version 10.0). Raw sequences were denoised using the DADA2 pipeline to remove chimeric reads and low-quality bases. Taxonomic annotation of ASVs was performed using the Naïve Bayes classifier in QIIME2 with the SILVA database (release 138.1) and a confidence threshold of 70%. The ASV data were normalized to the minimum number of reads using the *microeco* package [29] in R version 4.4.2, followed by subsequent analyses and visualization. Alpha diversity was analyzed to evaluate species diversity within samples, while Beta diversity was assessed using principal coordinate analysis (PCoA) to evaluate differences in species composition between samples. The statistical significance of Beta diversity clustering between groups was determined using a non-parametric permutation test (PERMANOVA). Differentially abundant taxa were identified using linear discriminant analysis effect size (LEfSe) analysis. Additionally, genus-level differences between the HFD and Wv2365 groups were further evaluated using Metastats analysis to identify representative taxa with statistically significant changes (*p* < 0.05).

### 2.8. Metabolites Analysis

Serum samples (50 μL per rat, 6 samples per group) were mixed with 300 μL methanol containing internal standards, centrifuged at 12,000 rpm for 10 min at 4 °C, and stored at −20 °C. After centrifugation, 200 μL of the supernatant was collected for analysis. Metabolites were analyzed using an Agilent 1290 Infinity LC system and an AB 6500+ QTRAP mass spectrometer under optimized gradient conditions, with multiple reaction monitoring (MRM) mode used for detection. QC samples were included to ensure data reliability. Metabolomic data were normalized using median normalization and auto-scaling. Partial least squares discriminant analysis (PLS-DA) was performed using the *ropls* package in R (version 4.4.2). Volcano plots were generated to visualize metabolites of interest, which were defined as those with |log₂FC| > 1, *p* < 0.05, and Variable importance in projection (VIP) > 1. These metabolites were further evaluated using one-way analysis of variance (ANOVA) on their raw concentration values to assess statistical differences across experimental groups.

### 2.9. Statistical Analysis

Statistical analyses were performed using GraphPad Prism 9.5.1. Data are presented as mean ± standard deviation (SD). A two-way repeated measures analysis of variance (two-way RM ANOVA) was used to assess body weight changes over time, with time and treatment as factors. The Geisser–Greenhouse correction was applied to adjust for violations of the sphericity assumption, and Dunnett’s multiple-comparisons test was used for post hoc analysis. For other quantitative data, appropriate statistical tests were selected based on data distribution and variance homogeneity. For normally distributed data with homogeneous variances, one-way ANOVA was used for multiple-group comparisons. When the assumption of homogeneity of variance was violated, Brown–Forsythe and Welch ANOVA were applied. For non-normally distributed data, the Kruskal–Wallis test followed by Dunn’s post hoc test was used. Multiple hypothesis testing corrections were performed using the Benjamini–Hochberg (BH) method to control the false discovery rate (FDR). A *p*-value < 0.05 was considered statistically significant.

## 3. Results

### 3.1. Effects of Wv2365 on Body Weight, Food Intake, and Hepatic Parameters in MASLD Rats

**Figure 1:**
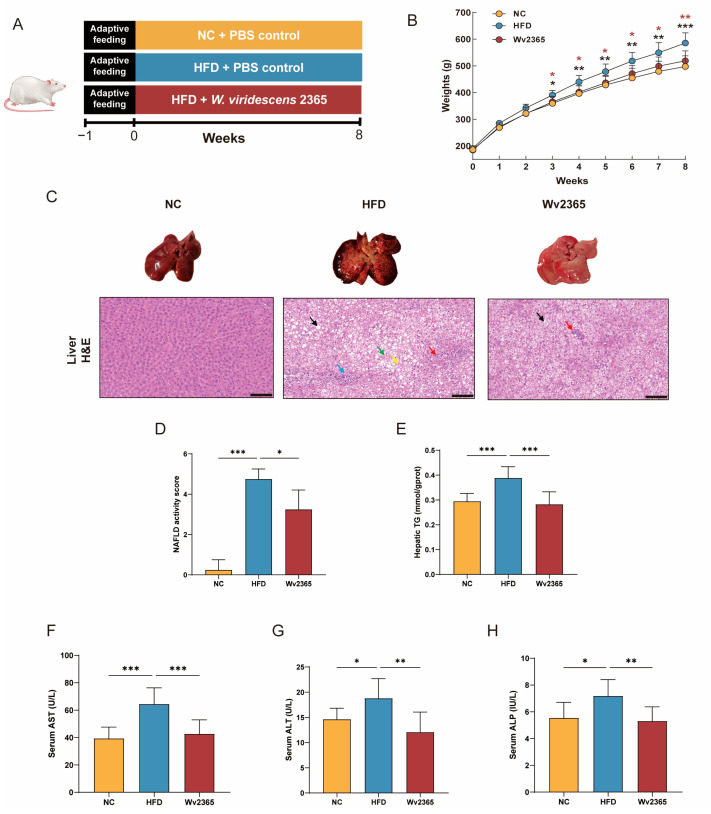
Wv2365 improves liver function. (**A**) Experimental design. (**B**) Body weight comparison among control (NC), high-fat diet (HFD), and HFD supplemented with Wv2365 (Wv2365) groups. (**C**,**D**) Representative H&E staining of liver sections and the corresponding NAS score. Histological features include large cytoplasmic vacuoles (black arrows), hepatocyte necrosis (yellow arrows), hemorrhage (green arrows), connective tissue proliferation (red arrows), and lymphocyte infiltration (blue arrows). Scale bar = 100 μm. (**E**) Hepatic TG content. (**F**–**H**) Serum levels of AST, ALT, and ALP. Results are expressed as mean ± SD. Statistical significance is indicated as * *p* < 0.05, ** *p* < 0.01, and *** *p* < 0.001.

To assess the protective effects of Wv2365 against HFD-induced MASLD rats, an 8-week intervention study was conducted (Figure 1A). From week 3, the weight of HFD group rats was significantly higher than that of the NC group rats, while the Wv2365 intervention significantly reduced HFD-induced weight gain and returned their weight to the level of the NC group (Figure 1B). Notably, food intake and energy intake remained comparable between the HFD and Wv2365 groups, indicating that the weight loss effect of Wv2365 was not due to reduced food consumption (Figure S1A–C).

H&E staining of liver sections showed that the liver structure was normal in the NC group, and severe hepatic steatosis, necrosis, and immune cell infiltration occurred in the HFD group, while Wv2365 supplementation significantly alleviated hepatic steatosis and inflammation caused by HFD. The NAS score showed that the Wv2365 group had a lower score than the HFD group, indicating that Wv2365 supplementation alleviated liver injury symptoms (*p* < 0.001; Figure 1C,D). Compared with the NC group, the levels of the hepatic TG and serum AST, ALT, and ALP in the HFD group were significantly increased, and which was reversed by the Wv2365 intervention (*p* < 0.001; Figure 1E–H). Consistently, the liver index (liver weight/body weight ratio), a common indicator of liver enlargement and lipid accumulation, was significantly higher in the HFD group compared with the NC group, while significantly lower in the Wv2365 group compared with the HFD group (*p* < 0.01; Figure S1D).

### 3.2. Effects of Wv2365 on Serum Lipid and Adipocyte Morphology in MASLD Rats

**Figure 2:**
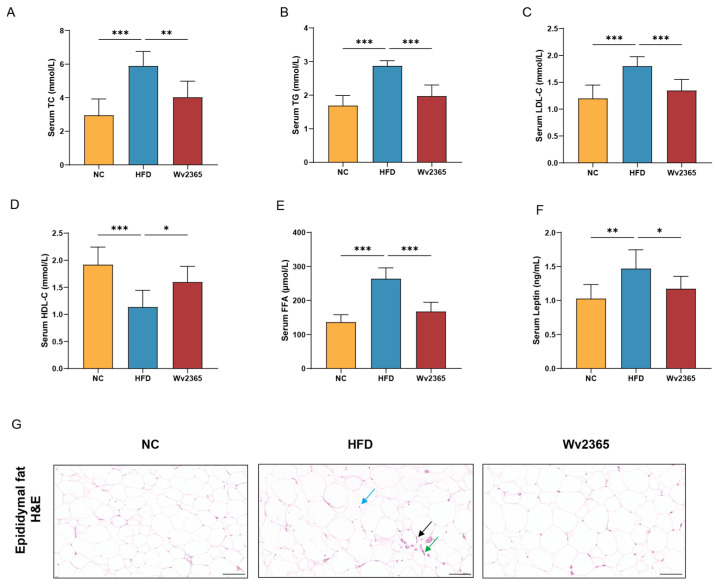
Wv2365 improves serum lipid and adipose tissue dysfunction. (**A**–**E**) Serum lipid profile across groups, including TC, TG, LDL-C, HDL-C, and FFA. (**F**) Serum leptin level reflecting adipose tissue function. (**G**) H&E staining of epididymal adipose tissue highlighting lymphocytes (black arrows), granulocytes (green arrows), and macrophages (blue arrows). Scale bar = 100 μm. Results are expressed as mean ± SD. Statistical significance is indicated as * *p* < 0.05, ** *p* < 0.01, and *** *p* < 0.001.

Serum TC, TG, LDL-C, leptin, and FFA levels in the HFD group were significantly higher than those in the NC group and Wv2365 groups (*p* < 0.05), and serum HDL-C levels were significantly lower than those in the NC group and Wv2365 groups (*p* < 0.05; Figure 2A–F).

A histological examination of adipose tissue revealed an intact adipocyte morphology in the NC group, whereas the HFD group exhibited pronounced immune cell infiltration, including lymphocytes, granulocytes, and macrophages. Wv2365 treatment significantly attenuated immune cell infiltration and preserved a normal adipose tissue structure (Figure 2G). Similarly, the epididymal fat index was significantly increased in the HFD group compared to the NC group, but significantly reduced in the Wv2365 group relative to the HFD group, indicating that Wv2365 could limit adipose tissue accumulation (*p* < 0.05; Figure S1E).

### 3.3. Wv2365 Modulates Inflammation-Related Hepatic Transcriptomic Profiles in MASLD Rats

**Figure 3:**
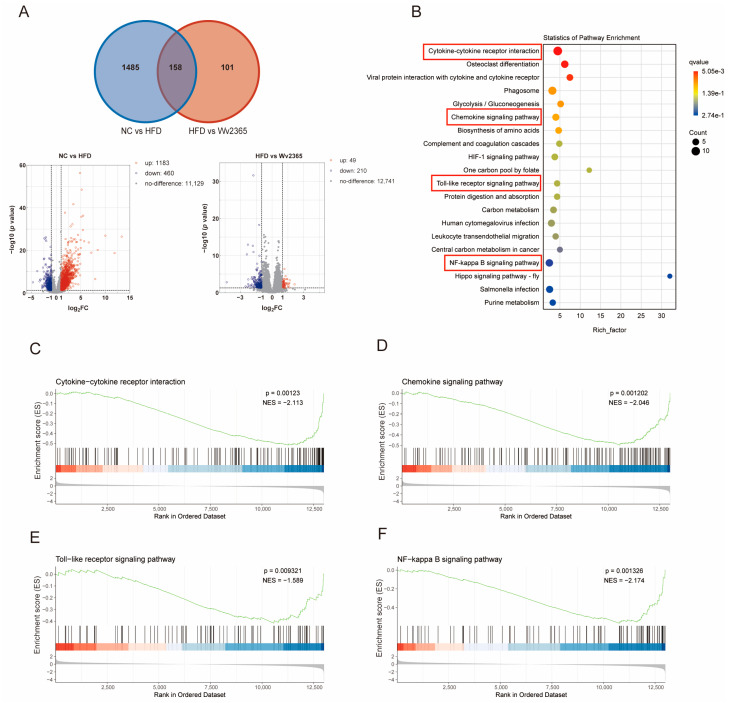
Transcriptomic analysis reveals Wv2365-mediated regulation of inflammatory pathways. (**A**) Venn diagram illustrating the overlap and unique DEGs among the three experimental groups. Volcano plot displaying DEGs between the NC vs. HFD and HFD vs. Wv2365 groups, highlighting upregulated (red) and downregulated (blue) genes. (**B**) Bubble plot representing enriched KEGG pathways among DEGs, with pathway significance indicated by bubble size and color. (**C**–**F**) GSEA analysis showing activation of key inflammatory pathways in the Wv2365 group compared to the HFD group.

Transcriptomic profiling of liver tissues identified 1183 upregulated and 460 downregulated genes in the HFD group relative to the control. In contrast, a comparison between the Wv2365 and HFD groups revealed 49 upregulated and 210 downregulated genes. The Venn diagram showed 158 overlapping DEGs across the three groups (Figure 3A). A KEGG enrichment analysis revealed that these overlapping DEGs were significantly enriched in inflammation-related pathways, including cytokine–cytokine receptor interaction, chemokine signaling, Toll-like receptor signaling, and NF-κB signaling (*p* < 0.05; Figure 3B). A gene set enrichment analysis (GSEA) further confirmed that these inflammatory pathways were upregulated in the HFD group compared with the control group, but were downregulated in the Wv2365 group compared with the HFD group (*p* < 0.05; Figure 3C–F and Figure S2A–D).

### 3.4. Effects of Wv2365 on Serum Inflammation in MASLD Rats

**Figure 4:**
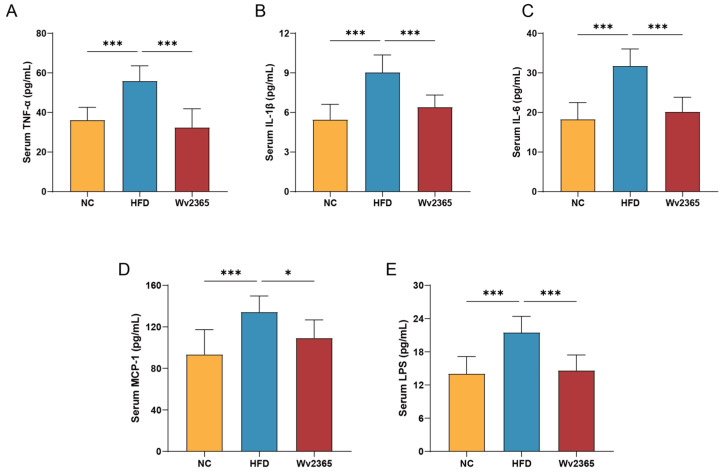
Wv2365 suppresses pro-inflammatory markers. (**A**–**E**) Serum levels of inflammatory cytokines and markers, including TNF-α, IL-1β, IL-6, MCP-1, and LPS, across experimental groups. Results are expressed as mean ± SD. Statistical significance is denoted as * *p* < 0.05 and *** *p* < 0.001.

Compared with the NC group, serum levels of TNF-α, IL-1β, IL-6, MCP-1, and LPS in the HFD group were significantly increased, and Wv2365 supplementation effectively reduced these levels (*p* < 0.05; Figure 4A–E).

### 3.5. Effects of Wv2365 on Oxidative Stress in MASLD Rats

**Figure 5:**
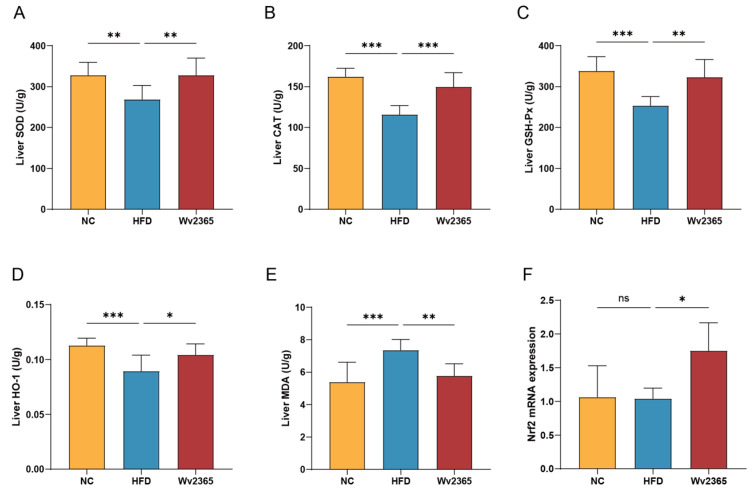
Wv2365 attenuates oxidative stress in the liver. (**A**–**D**) Hepatic antioxidant enzyme activities, including SOD, CAT, GSH-Px, and HO-1. (**E**) MDA levels. (**F**) The gene expression of *Nrf2*. Results are shown as mean ± SD. Statistical significance is indicated as * *p* < 0.05, ** *p* < 0.01, and *** *p* < 0.001.

Oxidative stress is a critical factor contributing to the progression of MASLD [30]. To evaluate the antioxidant response, key antioxidant enzymes and markers of oxidative damage were assessed. Hepatic activities of antioxidant enzymes, including SOD, CAT, GSH-Px, and HO-1, were significantly decreased in the HFD group (*p* < 0.01), whereas Wv2365 supplementation effectively restored their activities (*p* < 0.05; Figure 5A–D). Hepatic MDA levels was significantly elevated in the HFD group (*p* < 0.05) but was markedly reduced following Wv2365 intervention (*p* < 0.05; Figure 5E). As a master regulator of antioxidant defense, the transcription factor *Nrf2* plays a pivotal role in maintaining redox homeostasis [31]. Notably, the hepatic expression of *Nrf2* did not significantly differ between the NC and HFD groups but was significantly upregulated in the Wv2365-treated group (*p* < 0.05; Figure 5F).

### 3.6. Wv2365 Induces Gut Microbiota Alterations in MASLD Rats

**Figure 6:**
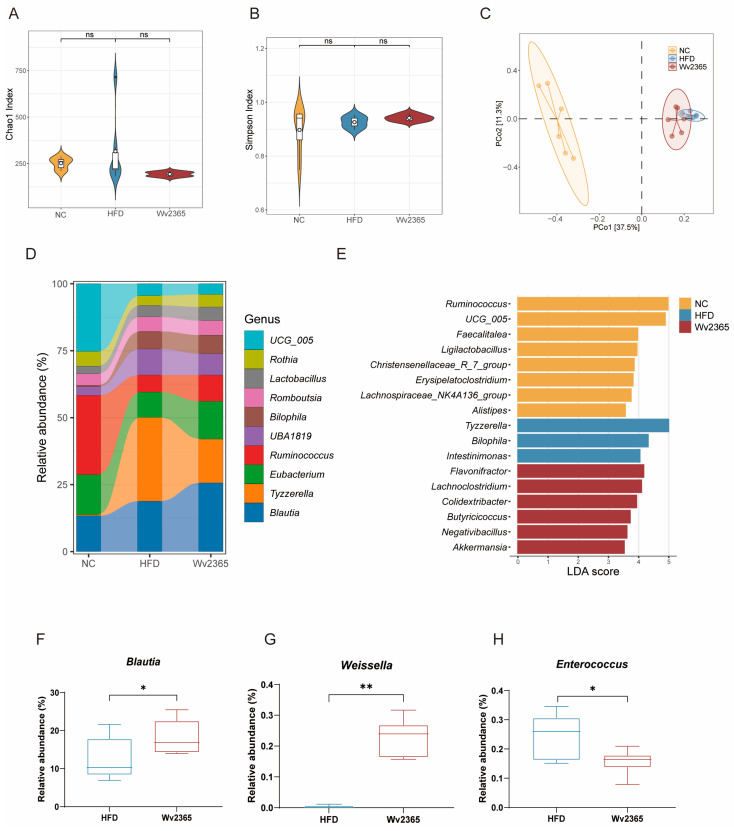
Wv2365 modulates gut microbiota composition. (**A**) Chao1 Index. (**B**) Simpson Index. (**C**) Beta diversity based on Bray–Curtis distances visualized by PCoA. (**D**) Genus-level relative abundance across groups. (**E**) Key microbial biomarkers identified by LEfSe (LDA > 3.5, FDR < 0.05). (**F**–**H**) Representative differentially abundant genera between HFD and Wv2365 groups identified by Metastats analysis, (**F**) *Blautia*, (**G**) *Weissella*, and (**H**) *Enterococcus*. * *p* < 0.05, ** *p* < 0.01.

16S rRNA sequencing revealed no significant differences in α-diversity among the three groups (Figure 6A,B). However, a β-diversity analysis using PCoA demonstrated distinct clustering patterns between the NC, HFD, and Wv2365 groups (Figure 6C), suggesting a shift in microbial composition following Wv2365 supplementation. At the genus level, *Ruminococcus* was predominant in the NC group, while *Tyzzerella* dominated in the HFD group. In contrast, the Wv2365-supplemented group exhibited increased abundances of Blautia (Figure 6D). An LEfSe analysis identified key genera that significantly differed among the three groups. The NC group was enriched in *Ruminococcus*, *Ligilactobacillus*, and *Alistipes*, while the HFD group exhibited increased levels of *Tyzzerella* and *Bilophila*. Wv2365 supplementation significantly increased the relative abundance of *Butyricicoccus* and *Akkermansia* (LDA score > 3.5; Figure 6E). To further validate the genus-level differences between the HFD and Wv2365 groups, a Metastats analysis revealed a significant increase in the relative abundances of *Blautia* and *Weissella* in the Wv2365 group, whereas the abundance of *Enterococcus* was significantly reduced (*p* < 0.05; Figure 6F–H).

### 3.7. Wv2365 Altered Serum Metabolites in MASLD Rats

**Figure 7:**
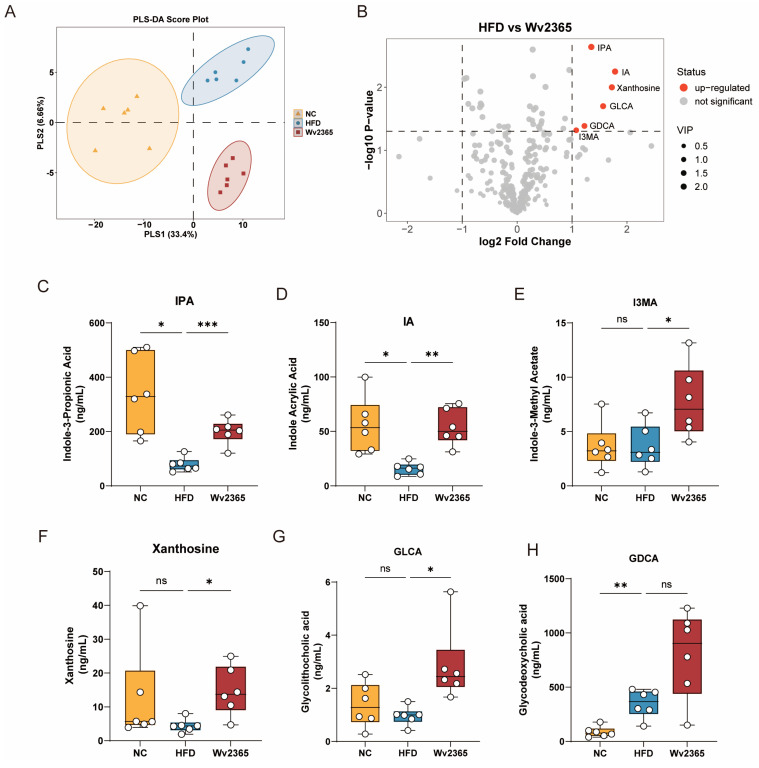
Wv2365 regulates serum metabolism. (**A**) PLS-DA score plot showing clear separation among NC, HFD, and Wv2365 groups. (**B**) Volcano plot of differential metabolites between the HFD and Wv2365 groups. Dot color indicates the direction of fold change: red represents significantly upregulated metabolites in the Wv2365 group, and grey indicates non-significant changes, based on thresholds of |log₂(FC)| > 1, VIP > 1, and *p* < 0.05. Dot size reflects the variable VIP score. (**C**–**H**) Serum concentrations of IPA, IA, I3MA, xanthosine, GLCA, and GDCA. Data are presented as box plots showing the median and interquartile range (n = 6 per group). Statistical analysis was performed using one-way ANOVA. * *p* < 0.05, ** *p* < 0.01, and *** *p* < 0.001; ns, not significant.

A total of 358 serum metabolites, including lipids, amino acids, carbohydrates, and bile acids, were identified by LC-MS/MS-based wide-targeted metabolomics. PLS-DA revealed distinct separations among the NC, HFD, and Wv2365 groups (Figure 7A), indicating substantial differences in metabolic profiles. Six metabolites were significantly upregulated in the Wv2365 group compared to the HFD group, as illustrated in the volcano plot (Figure 7B). In contrast, the volcano plot for the NC vs. HFD comparison (Figure S3A) identified 44 differential metabolites, including 12 upregulated and 32 downregulated compounds. One-way ANOVA based on raw concentration values confirmed that several metabolites exhibited statistically significant changes. Compared with the NC group, the HFD group showed significantly reduced levels of IPA and IA, while I3MA, xanthosine, and GLCA remained unchanged. In addition, glycodeoxycholic acid (GDCA) was significantly elevated in the HFD group. Following Wv2365 supplementation, the levels of IPA, IA, I3MA, xanthosine, and GLCA were significantly increased compared with the HFD group (*p* < 0.05; Figure 7C–G), whereas GDCA levels showed no significant difference (Figure 7H).

## 4. Discussion

**Figure 8:**
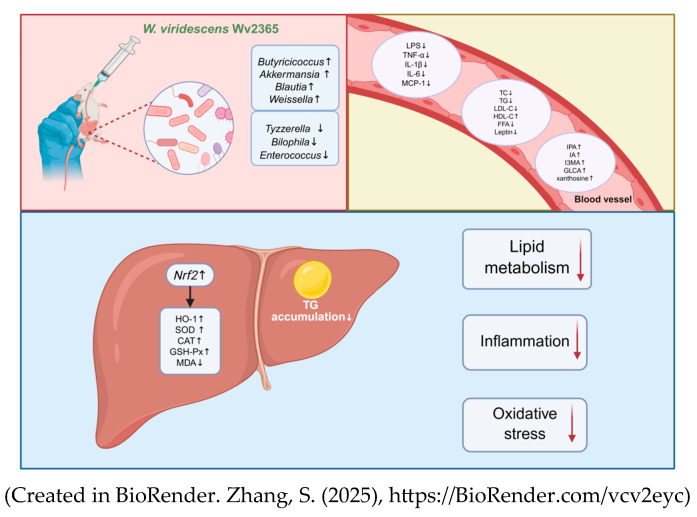
Proposed mechanisms by which *W. viridescens* Wv2365 alleviates MASLD. Oral administration of Wv2365 modulates gut microbiota composition, reduces inflammatory markers, improves lipid profiles, and enhances serum metabolic profiles. In the liver, Wv2365 activates the Nrf2/HO-1 pathway, increases antioxidant enzyme activity, and reduces triglyceride accumulation. These effects collectively improve lipid metabolism, attenuate inflammation, and alleviate oxidative stress.

*Weissella* species have attracted increasing scientific interest due to their probiotic potential and beneficial metabolic effects [23]. For example, *W. cibaria* MG5285 was shown to reduce adipose fat accumulation and hepatic steatosis in high-fat-diet-induced obese mice [32], suggesting that certain strains in this genus may alleviate metabolic disorders. Despite these findings, *W. viridescens* has been mostly studied for its immunomodulatory activity, and its potential role in metabolic disease remains unclear. This study evaluated the protective effects of *W. viridescens* Wv2365 in an HFD-induced rat model of MASLD. Wv2365 supplementation significantly alleviated hepatic steatosis, improved lipid metabolism, reduced systemic inflammation, and enhanced antioxidant defenses. These findings suggest that Wv2365 exerts its hepatoprotective effects through a multifaceted mechanism involving the modulation of inflammatory and oxidative stress pathways, the remodeling of gut microbiota composition, and the regulation of host metabolic responses (Figure 8).

Metabolic inflammation is a hallmark of HFD-induced MASLD and is primarily attributed to a disrupted lipid metabolism and the dysbiosis of the gut microbiota [33,34]. Elevated levels of circulating FFAs lead to excessive hepatic TG accumulation, thereby aggravating oxidative stress and triggering chronic low-grade systemic inflammation [35]. Meanwhile, gut microbiota dysbiosis promotes intestinal permeability and facilitates the translocation of LPS into the systemic circulation, further exacerbating inflammatory responses [36,37]. As a key microbial-derived endotoxin, LPS activates pattern recognition receptors such as Toll-like receptors, thereby initiating the NF-κB signaling pathway. This inflammatory cascade has been widely documented in hepatic tissues of MASLD patients and animal models [38,39]. In the current study, Wv2365 supplementation significantly reduced serum LPS levels and downregulated key inflammatory pathways, including Toll-like receptor signaling and NF-κB signaling. Notably, the serum levels of pro-inflammatory cytokines such as TNF-α, IL-1β, IL-6, and MCP-1 were markedly decreased. These findings suggest that Wv2365 may attenuate inflammation by suppressing inflammation-related signaling cascades.

Oxidative stress is another key contributor to the progression of MASLD, as it promotes lipid peroxidation, mitochondrial dysfunction, and hepatocyte apoptosis [30,40]. The *Nrf2* signaling pathway plays a pivotal role in the cellular defense against oxidative stress. *Nrf2* is a cytoplasmic transcription factor that regulates the expression of various antioxidant enzymes. Under normal physiological conditions, *Nrf2* remains bound in the cytoplasm by Kelch-like ECH-associated protein 1 (Keap1), which promotes its ubiquitination and subsequent degradation via the proteasome. Upon oxidative stress, *Nrf2* dissociates from Keap1, translocates into the nucleus, and interacts with antioxidant response elements, thereby initiating the transcription of antioxidant enzymes, including SOD, CAT, GSH-Px, and HO-1 [41,42]. These enzymes are essential for neutralizing reactive oxygen species, reducing lipid peroxidation, and preserving cellular integrity [43,44]. In the present study, Wv2365 supplementation significantly upregulated hepatic *Nrf2* expression and enhanced antioxidant capacity, as evidenced by elevated activities of antioxidant enzymes and decreased levels of MDA.

Beyond its classical antioxidative role, *Nrf2* also modulates inflammatory responses through multiple mechanisms. It antagonizes the NF-κB pathway by competing for transcriptional coactivators such as CREB-binding protein, thereby limiting the NF-κB-driven cytokine expression [45]. Moreover, HO-1, a key downstream target of *Nrf2*, acts as a central hub linking redox homeostasis and immune regulation. In addition to its role in degrading pro-oxidant heme, HO-1 exerts potent anti-inflammatory effects by suppressing the production of pro-inflammatory cytokines such as TNF-α, IL-1β, and IL-6, promoting M2 macrophage polarization, and inhibiting NF-κB activation [46]. The concomitant upregulation of *Nrf2* and HO-1 in Wv2365-treated rats may, therefore, underlie the observed attenuation of both oxidative stress and inflammatory signaling. Furthermore, *Nrf2* regulates mitochondrial function by promoting mitochondrial biogenesis, enhancing mitochondrial antioxidant defenses, and preventing membrane depolarization [47]. These actions collectively preserve mitochondrial integrity and sustain the cellular energy balance, thereby protecting against hepatocyte apoptosis and halting MASLD progression.

In addition to its molecular effects within the liver, Wv2365 supplementation was associated with systemic metabolic improvements. Specifically, Wv2365 treated rats exhibited significant weight reduction without differences in food or energy intake, indicating enhanced metabolic efficiency. This was accompanied by reduced hepatic triglyceride levels, lower circulating FFAs, a smaller adipocyte size, and decreased serum leptin concentrations, suggesting improved lipid mobilization, adipose tissue remodeling, and reduced adipose-derived inflammatory signaling. Given that adipose tissue dysfunction contributes to MASLD by promoting lipolysis, increasing FFA flux to the liver, and releasing pro-inflammatory adipokines such as TNF-α, IL-6, and leptin [48], these changes imply that Wv2365 may exert beneficial regulatory effects on the liver–adipose tissue axis. These findings suggest that Wv2365 may modulate MASLD progression by improving adipose tissue metabolism and its crosstalk with the liver.

The progression of MASLD is closely linked to alterations in the gut microbiota composition. Previous studies, including that of Hu et al., have reported that increased abundances of *Tyzzerella* and *Bilophila* are significantly associated with MASLD phenotypes [49]. In our study, these taxa were also enriched in the HFD group. Notably, Wv2365 supplementation modulated the gut microbiota composition by enriching several beneficial bacterial genera, including *Akkermansia*, *Butyricicoccus*, and *Blautia*. *Akkermansia* is well-known for enhancing the intestinal barrier integrity and reducing endotoxemia, thereby contributing to improved metabolic homeostasis [50,51]. In particular, *A. muciniphila* has been linked to enhanced lipid oxidation and improved gut–liver axis function through microbiota remodeling [52,53]. *Butyricicoccus*, a known butyrate producer, has been shown to enhance epithelial barrier function and support intestinal homeostasis [54]. Moreover, the oral administration of *Blautia wexlerae* has been shown to promote metabolic remodeling and exert anti-inflammatory effects, alleviating HFD-induced obesity and insulin resistance [55]. In addition, *B. coccoides* has demonstrated the ability to metabolize tryptophan into indole-3-acetic acid (I3AA), which activates the aryl hydrocarbon receptor in the liver and exerts anti-inflammatory effects [56]. Consistent with these findings, Wv2365 intervention significantly enriched *Akkermansia*, *Butyricicoccus*, and *Blautia*, while reducing the abundance of potentially pathogenic genera such as *Enterococcus*. These results suggest that Wv2365 may mitigate MASLD by reshaping the gut microbiota toward a more healthiness-associated profile, thereby contributing to improved metabolic regulation and attenuation of inflammation.

Alterations in the gut microbiota composition and function are often accompanied by changes in host metabolite profiles, which may mediate the beneficial effects of probiotic interventions on metabolic health. In this study, metabolomic profiling revealed significant increases in several bioactive serum metabolites following Wv2365 supplementation, including IPA, IA, I3MA, GLCA, and xanthosine. Among these, IPA, IA, and I3MA are indole derivatives produced through gut-microbiota–mediated tryptophan metabolism. Indole and its derivatives are well-known microbial metabolites with critical roles in modulating host immune and inflammatory responses [57,58]. IPA has been shown to confer hepatoprotective effects by reducing oxidative stress and inflammation in MASLD models [59]. Mechanistically, IPA supplementation suppresses NF-κB signaling by lowering endotoxin levels and inhibiting macrophage activation [60]. IA also exhibits anti-inflammatory and antioxidant activities in LPS-stimulated human peripheral blood mononuclear cells, characterized by reduced IL-6 and IL-1β secretion and activation of the Nrf2-ARE pathway [61]. I3MA, another tryptophan-derived microbial metabolite, has been associated with immune regulation, and its levels are diminished in inflammatory conditions such as pediatric-enthesitis-related arthritis, a form of inflammatory bowel disease [62,63]. The elevation of GLCA, a gut-microbiota-derived secondary bile acid, has been associated with TGR5 activation, which may mediate its anti-inflammatory and metabolic effects [64]. Additionally, xanthosine has been reported to regulate glucose metabolism by inhibiting hepatic gluconeogenesis and promoting glycogenesis via the activation of the AMPK/FoxO1 and AKT/GSK3β pathways [65]. These metabolite alterations suggest that host metabolic responses are regulated by Wv2365 via multiple microbiota–host interaction pathways, potentially contributing to its protective effects against MASLD.

## 5. Conclusions

In conclusion, Wv2365 has been shown to exert multiple beneficial effects against MASLD, including the regulation of lipid metabolism, inflammation, and oxidative stress, as well as the modulation of the gut microbiota composition and improvement in serum metabolic pathways. Our findings highlight the potential of Wv2365 as a probiotic candidate for the comprehensive modulation of MASLD through a multifactorial mechanism.

## Data Availability

All sequencing data are publicly available in the NCBI SRA database (BioProject numbers: PRJNA1119725).

## Supplementary Materials

The following supporting information can be downloaded at: https://www.mdpi.com/article/10.3390/nu17091585/s1, Figure S1: Wv2365 attenuates weight gain, increases food intake efficiency, and reduces fat accumulation in MASLD rats; Figure S2: GSEA of inflammatory pathways activated in the HFD group compared to NC; Figure S3: Volcano plot showing differential serum metabolites between NC and HFD groups.

## Abbreviations

The following abbreviations are used in this manuscript:

MASLD	Metabolic-dysfunction-associated steatotic liver disease
NAS	NAFLD Activity Score
NC	Normal chow diet
HFD	High-fat diet
PBS	Phosphate-buffered saline
TC	Total cholesterol
TGs	Triglycerides
LDL-C	Low-density lipoprotein cholesterol
HDL-C	High-density lipoprotein cholesterol
FFAs	Free fatty acids
ALT	Alanine aminotransferase
AST	Aspartate aminotransferase
ALP	Alkaline phosphatase
LPSs	Lipopolysaccharides
TNF-α	Tumor necrosis factor-alpha
IL-1β	Interleukin-1 beta
IL-6	Interleukin-6
MCP-1	Monocyte Chemoattractant Protein-1
SOD	Superoxide dismutase
CAT	Catalase
GSH-Px	Glutathione peroxidase
HO-1	Heme oxygenase-1
MDA	Malondialdehyde
*Nrf2*	Nuclear factor erythroid 2–related factor 2
IPA	Indole-3-propionic acid
IA	Indoleacrylic acid
GLCA	Glycolithocholic acid
