# Supplementary figures and images for: Weissella viridescens Attenuates Hepatic Injury, Oxidative Stress, and Inflammation in a Rat Model of High-Fat Diet-Induced MASLD
Source: Nutrients. 2025 May 5;17(9):1585. doi: 10.3390/nu17091585 (PMC12073722; doi:10.3390/nu17091585)

A

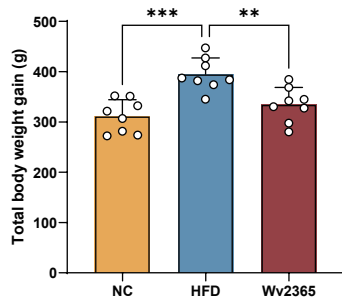

B

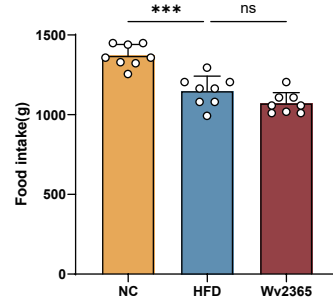

C

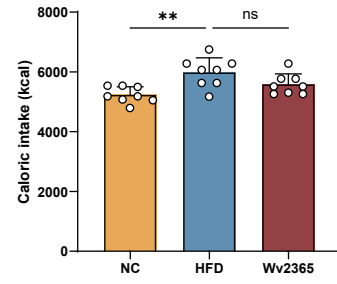

D

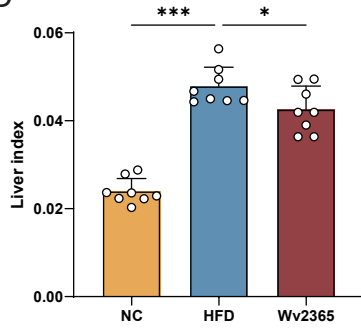

E

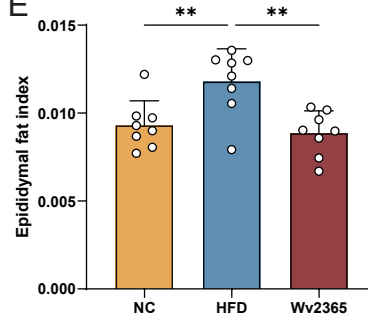

Supplement: Supplementary file 1 [file nutrients-17-01585-s001.zip › Figure S1 Wv2365 attenuates weight gain, increases food intake efficiency, and reduces fat accumulation in MASLD rats.pdf]

A

NC vs HFD

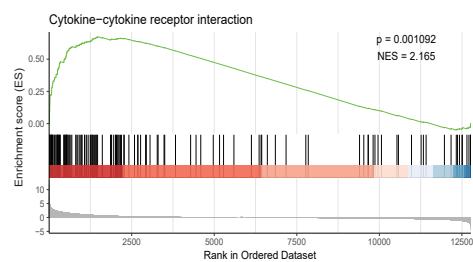

B

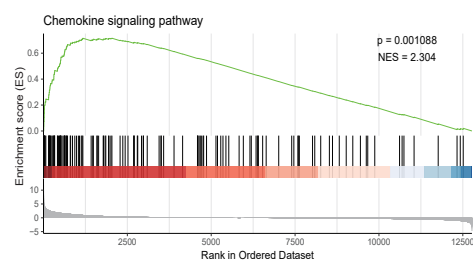

C

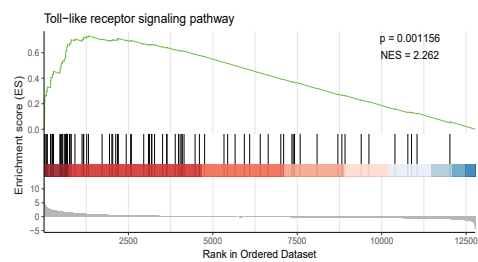

D

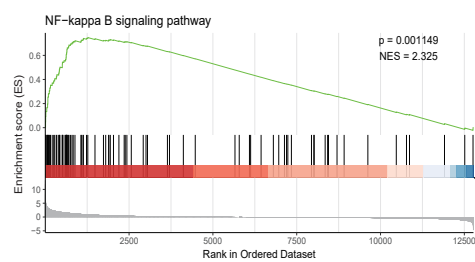

Supplement: Supplementary file 1 [file nutrients-17-01585-s001.zip › Figure S2 GSEA of inflammatory pathways activated in the HFD group compared to NC.pdf]

A

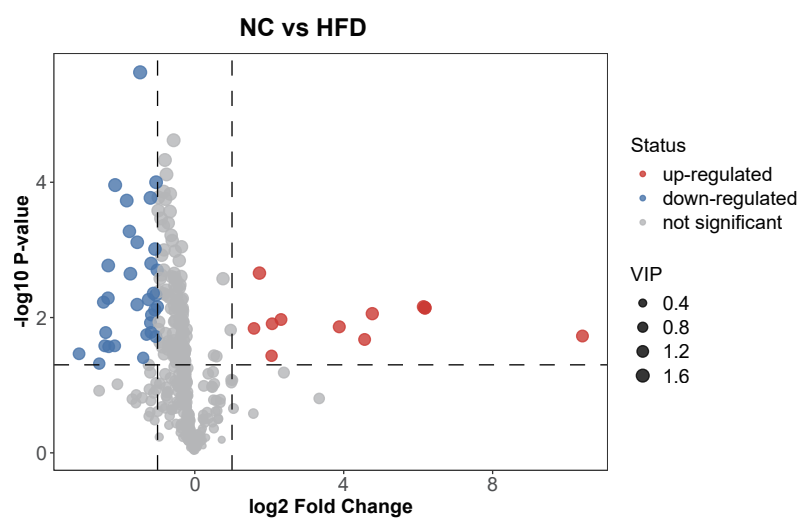

Supplement: Supplementary file 1 [file nutrients-17-01585-s001.zip › Figure S3 Volcano plot showing differential serum metabolites between NC and HFD groups.pdf]
